# Supplementary figures and images for: Clinical implication and potential function of ARHGEF6 in acute myeloid leukemia: An in vitro study
Source: PLoS One. 2023 Apr 7;18(4):e0283934. doi: 10.1371/journal.pone.0283934 (PMC10081785; doi:10.1371/journal.pone.0283934)

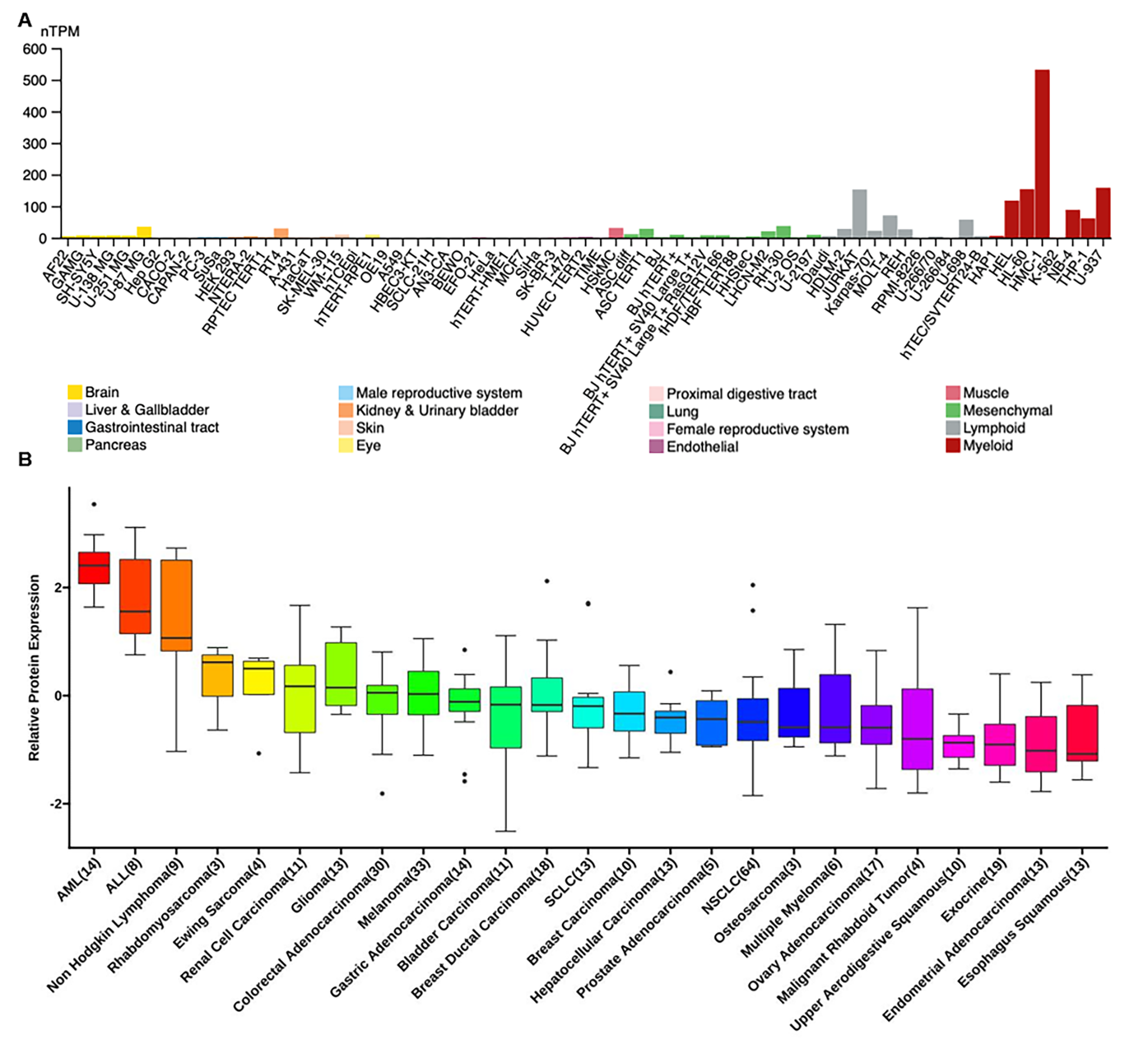

Supplement: S1 Fig — (A) The mRNA levels of ARHGEF6 in human cell lines using the HPA. (B) The relative protein levels of ARHGEF6 in human cell lines using the CCLE. Labels of x-axis were sorted from large to small according to the median expression of protein. (TIF) [file pone.0283934.s001.tif]
