# Supplementary material for: Clinical implication and potential function of ARHGEF6 in acute myeloid leukemia: An in vitro study
Source: PLoS One. 2023 Apr 7;18(4):e0283934. doi: 10.1371/journal.pone.0283934 (PMC10081785; doi:10.1371/journal.pone.0283934)
Supplement: Attachment — Submitted filename: Response to Reviewers.docx [file pone.0283934.s006.docx]

Dear Dr. Senthilnathan Palaniyandi,

Thank you for your letter dated January 12. We were pleased to know that our work was rated as potentially acceptable for publication in ***PLOS ONE***, subject to adequate revision. We thank the reviewers for the time and effort that they have put into reviewing the previous version of the manuscript. Their suggestions have enabled us to improve our work. Based on the instructions provided in your letter, we uploaded the file of the revised manuscript. Accordingly, we have uploaded a copy of the original manuscript with all the changes highlighted by using the track changes mode in MS Word. Appended to this letter is our point-by-point response to the comments raised by the reviewers. The comments are reproduced and our responses are given directly afterward. We would like also to thank you for allowing us to resubmit a revised copy of the manuscript.

We hope that the revised manuscript is accepted for publication in ***PLOS ONE***.

Sincerely,

Jiankui Zhou

Encl. Responses to the comments from Reviewers #1 and #2.

Reply to Reviewer #1

Dear Reviewer,

Thank you very much for your time involved in reviewing the manuscript; your responsible attitude toward patients is admirable.

Comments:

“This article is bioinformatic and it just collected published data and then analyzed them. Most of data derived from cell lines, the experiment result of cell line are artificial data not real data. we discuss on AML it meaning patients life, by statically analysis we can reach to use one drug is good or not. Any Patient is unique.”

We also appreciate your clear and detailed feedback and hope that the explanation has fully addressed all of your concerns.

In this work, we focused on the expression characteristics, clinical prognosis, and potential function of the Rho guanine nucleotide exchange factor6 (ARHGEF6) in acute myeloid leukemia (AML). Through analysis of multiple databases (2 datasets on cell line; 4 datasets on RNA levels of patients; 2 datasets on patients’ clinical records), we found that:

(1) As a member of Rho guanine nucleotide exchange factors (GEFs), ARHGEF6 expression in AML has not been reported. By analyzing several databases, we found that ARHGEF6 is highly expressed in tissues and cell lines of AML. Pan-cancer analysis of ARHGEF6 showed its highest expression in AML. In the analysis of several common AML recurrence-associated karyotypes, we found that ARHGEF6 expression was significantly higher in t(8;21) compared to other karyotypes.
(2) Previous studies have generally concluded that Rho GEFs play a role in cancer promotion because they activate Rho GTPases. But a large part of Rho GEFs has not been sufficiently studied in various cancer types. Our analysis of the TCGA database and LinkedOmics database revealed that overall survival was significantly higher in patients with high ARHGEF6 expression, suggesting that overexpression of ARHGEF6 is associated with a good prognosis in AML. Besides, low ARHGEF6 expression patients can benefit from auto/allo-HSCT. These results can guide clinical treatment.
(3) ARHGEF6 downregulates the expression level of HOX family genes which inhibit hematopoietic stem cell differentiation, meanwhile increasing the expression of TRH, a favorable prognostic factor in AML. These results somewhat corroborate our previous findings and reveal the role of ARHGEF6 in AML.

Although we used data from cell lines, this part of the data only presented in FigS1, and all data presented in the text are from AML patients.

**Fig 1. ARHGEF6 expression in AML.** (A) ARHGEF6 expression in pan-cancer, result from UALCAN. (B) The expression of ARHGEF6 in different cancerous and normal tissues using the GEPIA. Bar height refers to the value of the median expression. (C) ARHGEF6 expression between AML and normal samples, result was obtained by GEPIA analysis. *, p < 0.05. (D) ARHGEF6 expression in AML among various karyotypes, according to GSE14468 in the GEO database. ****, p < 0.0001.

**Fig 2. Correlation between AML patient survival and ARHGEF6 expression with different factors.** (A) The prognostic value between ARHGEF6^low^ and ARHGEF6^high^ group using the LinkedOmics database. (B) OS and (C) DFS in AML patients with different ARHGEF6 expressions. (D) OS and (E) DFS in CN-AML patients with different ARHGEF6 expressions. (F) OS and (G) DFS of ARHGEF6^low^ and ARHGEF6^high^ patients treated with chemotherapy. (H) OS and (I) DFS of ARHGEF6^low^ and ARHGEF6^high^ patients undergoing chemotherapy + auto/allo-HSCT. (J) OS and (K) DFS of patients treated WOW auto/allo-HSCT in the ARHGEF6^low^ group.

**Fig 3. Functional annotation and prognosis validation of differentially expressed genes between ARHEGF6^low^ and ARHGEF6^high^ group.** (A) Volcano plot of DEGs between the ARHGEF6^low^ and ARHGEF6^high^ groups. (B) GO analysis of significantly upregulated genes. (C) GO analysis of significantly downregulated genes. (D) KEGG analysis of DEGs. (E) and (F) TRH expression levels in AML and normal tissues, and the effect on OS in AML patients, result from GEPIA. *, p < 0.05. (G) and (H) HOXA9 expression levels in AML and normal tissues, and the effect on OS in AML patients, result from GEPIA. *, p < 0.05. (I) and (J) HOXB6 expression levels in AML and normal tissues, and the effect on OS in AML patients, result from GEPIA. *, p < 0.05.

**Table 1. Correlations between ARHGEF6 expression and clinicopathological characteristics in AML from the TCGA cohort**

*n* number of patients, *WHO* World Health Organization, *AML-MRC* AML with myelodysplasia-related changes, *t-AML* Therapy-related AML, *NOS* not otherwise specified, *BM-blast* bone marrow blast, *WBC* white blood cell, *PB-blast* peripheral blood blast

Providing treatment plans to patients need to be personalized, and we should be responsible for the patient's life. Basic research is a long process from bench to bedside. Research starts from the cellular level, then move up to the animal, and finally is validated with precious clinical patient samples. For humans, the number of genes to be studied is massive, and screen for some genes with key roles in a limited time is crucial. Bioinformatic analysis can accelerate this process and guide subsequent laboratory and clinical studies.

We would like to take this opportunity to thank you for all your time involved and this great opportunity for us to improve the manuscript. We hope you will find this revised version satisfactory.

Sincerely,

Jiankui Zhou

-----End of Reply to Reviewer #1------

Reply to Reviewer #2

Dear Dr. Supriya Gupta,

Thank you very much for your time in reviewing the manuscript and your encouraging comments on the merits.

Comments:

“The authors have highlighted an important finding of high ARHGEF6 expression in patients with AML being associated with a favorable prognosis and low expression being associated with a poor prognosis. The authors have also highlighted the improvement of outcomes by using hematopoietic stem cell transplantation in patients with ARHGEF6 low expression. Additionally, the role of the HOX gene family and TRH has been highlighted which is an important prognostic finding. The paper is well written, informative and I believe will be valuable to readers given its scientific merit.”

We also appreciate your clear and detailed feedback and hope that the explanation has fully addressed all of your concerns. In the remainder of this letter, we discuss each of your comments individually along with our corresponding responses.

Comment1:

“The only point I would like to make is that FAB classification of AML is now obsolete and it would be interesting to see the trends of ARHGEF6 expression with the new classification.”

Response1:

Thanks for your great suggestion on improving the accessibility of our manuscript. We have re-analyzed the data using the WHO classification published in 2016. The relevant contents are provided below as a screen dump for your quick reference.

**Table 1. Correlations between ARHGEF6 expression and clinicopathological characteristics in AML from the TCGA cohort**

*n* number of patients, *WHO* World Health Organization, *AML-MRC* AML with myelodysplasia-related changes, *t-AML* Therapy-related AML, *NOS* not otherwise specified, *BM-blast* bone marrow blast, *WBC* white blood cell, *PB-blast* peripheral blood blast

The WHO classification and risk stratification were significantly different in patients with different ARHGEF6 expressions. In the WHO classification distribution, high ARHGEF6 expression was significantly associated with genetic abnormalities.

We would like to take this opportunity to thank you for all your time involved and this great opportunity for us to improve the manuscript. We hope you will find this revised version satisfactory.

Sincerely,

Jiankui Zhou

-----End of Reply to Reviewer #2------
