# Supplementary material for: Clinical implication and potential function of ARHGEF6 in acute myeloid leukemia: An in vitro study
Source: PLoS One. 2023 Apr 7;18(4):e0283934. doi: 10.1371/journal.pone.0283934 (PMC10081785; doi:10.1371/journal.pone.0283934)
Supplement: Attachment — Submitted filename: Response to Reviewers.docx [file pone.0283934.s007.docx]

Dear Dr. Senthilnathan Palaniyandi,

Thank you for your letter dated March 9. We are pleased to know that our work was rated as potentially acceptable for publication in ***PLOS ONE***, subject to adequate revision. We thank the reviewer for the time and effort in reviewing the previous version of the manuscript. The suggestions have enabled us to improve our work. Based on the instructions provided in your letter, we revised the article title and uploaded the file of the revised manuscript. Accordingly, we have uploaded a copy of the original manuscript with all the changes highlighted by using the track changes mode in MS Word. Appended to this letter is our response to the comment raised by the reviewer. We would like also to thank you for allowing us to resubmit a revised copy of the manuscript.

We hope that the revised manuscript is accepted for publication in ***PLOS ONE***.

Sincerely,

Zhou Jiankui

Encl. Responses to the comments from Reviewer #1.

Reply to Reviewer #1

Dear Reviewer,

Thank you very much for your time involved in reviewing the manuscript. Your suggestions are of great help to us.

Comments:

“I read the answers of correspond auteur, it described very well. as I suggest in last review. Same experiment is needed on primary AML cells derived from patients in two groups: M3 and non M3 groups. Or change the title : added in vitro results”

We also appreciate your clear and detailed feedback. In this revised version we have changed the title to “**Clinical implication and potential function of ARHGEF6 in acute myeloid leukemia: an *in vitro* study**”.

We would like to take this opportunity to thank you for all your time involved and this great opportunity for us to improve the manuscript. We hope you will find this revised version satisfactory.

Sincerely,

Zhou Jiankui

-----End of Reply to Reviewer #1------
